# Fishers (*Pekania pennanti*) are forest structure specialists when resting and generalists when moving: behavior influences resource selection in a northern Rocky Mountain fisher population

**DOI:** 10.1186/s40462-024-00487-5

**Published:** 2024-07-06

**Authors:** Lucretia E. Olson, Joel D. Sauder, Patrick A. Fekety, Jessie D. Golding, Carly W. Lewis, Rema B. Sadak, Michael K. Schwartz

**Affiliations:** 1 Rocky Mountain Research Station, United States Forest Service, Missoula, MT USA; 2 Idaho Department of Fish and Game, Lewiston, ID USA; 3 Natural Resource Ecology Laboratory, Colorado State University, Fort Collins, CO USA; 4 School of Natural Resources and the Environment, University of Arizona, Tucson, AZ USA; 5 Montana Ecological Services Field Office, United States Fish and Wildlife Service, Missoula, MT USA; 6 U.S. Department of Agriculture Forest Service, Intermountain Region, Ogden, UT USA

**Keywords:** Multi-scale habitat selection, LiDAR data, Fisher, *Pekania pennanti*, Forest management, Foraging habitat, Movement behavior

## Abstract

**Background:**

Studies of animal habitat selection are important to identify and preserve the resources species depend on, yet often little attention is paid to how habitat needs vary depending on behavioral state. Fishers (*Pekania pennanti*) are known to be dependent on large, mature trees for resting and denning, but less is known about their habitat use when foraging or moving within a home range.

**Methods:**

We used GPS locations collected during the energetically costly pre-denning season from 12 female fishers to determine fisher habitat selection during two critical behavioral activities: foraging (moving) or resting, with a focus on response to forest structure related to past forest management actions since this is a primary driver of fisher habitat configuration. We characterized behavior based on high-resolution GPS and collar accelerometer data and modeled fisher selection for these two behaviors within a home range (third-order selection). Additionally, we investigated whether fisher use of elements of forest structure or other important environmental characteristics changed as their availability changed, i.e., a functional response, for each behavior type.

**Results:**

We found that fishers exhibited specialist selection when resting and generalist selection when moving, with resting habitat characterized by riparian drainages with dense canopy cover and moving habitat primarily influenced by the presence of mesic montane mixed conifer forest. Fishers were more tolerant of forest openings and other early succession elements when moving than resting.

**Conclusions:**

Our results emphasize the importance of considering the differing habitat needs of animals based on their movement behavior when performing habitat selection analyses. We found that resting fishers are more specialist in their habitat needs, while foraging fishers are more generalist and will tolerate greater forest heterogeneity from past disturbance.

## Background

Understanding the resource needs of animals is fundamental to conservation biology, characterizing how an animal interacts with a landscape and informing management actions for conservation. An animal’s behavior can impact interactions with resources in ways that scientists often overlook [1]. As animals move throughout a landscape, the distribution of items necessary for an animal’s survival (i.e., resource needs) frequently requires them to accept tradeoffs between necessities such as food, water, or cover [2]. Resource selection occurs as individuals make movement choices based on the resources they encounter and their behavioral needs at the time [3]. Failure to account for the influence of behavior on resource selection can lead to wrong conclusions about the importance of certain resources [2], which can lead to conservation actions that may not accomplish conservation goals.

While behavior impacts the resource selection of animals, it has been frequently overlooked in resource selection studies, often due to difficulty in determining the behavioral state from common GPS location methods [4], although recent advancements in accelerometer technology [5] and behavioral state modeling [6, 7] have made it easier to determine and include behavioral state in studies of resource selection [8]. To reduce complexity, studies commonly categorize a small number of behavioral states, such as foraging, traveling, or resting, based on movement speed or tortuosity [5, 9]. These behavioral states measured with movement have been shown to impact how animals select resources, such as a foraging individual choosing areas with greater food availability [10] or a traveling individual selecting roads for faster movement [11]. The role of behavior in animal resource selection may be particularly important for conservation-relevant behaviors or life stages that may be more limiting with respect to habitat selection than others. Nesting or denning, for instance, often require a specific set of conditions, such as low disturbance [12] or structures for security [13], while traveling or fast-moving animals may be more tolerant to human disturbance [14, 15] or a wider range of habitat conditions [16, 17].

Habitat selection analyses are a useful tool to understand the environmental associations of a species, but they are subject to the assumption that selection or avoidance of a resource is consistent across individuals, while often, an animal’s selection or avoidance of a resource depends on how much of the resource is available to them [4]. This differential selection of resources in response to their availability is known as a functional response [18]. Functional responses in habitat selection are inferred by looking for patterns in use or selection among individuals across a gradient of resource availability. Deviations from consistent proportional use compared to availability indicate different habitat selection strategies. A classic example is a habitat tradeoff, in which selection is high for a given resource when availability is low (for example, a squirrel selecting open areas to forage in when forest cover is abundant and open areas are rare) but the resource becomes increasingly avoided as availability increases (the squirrel avoids open areas when they are more abundant than forest cover; [18]).

Generalist or specialist habitat selection can also be inferred from the presence of a functional response; specialists show stronger selection for an important resource when availability is low, while generalists lack a functional response and show proportional selection of a resource regardless of its availability [19]. Furthermore, the selectivity of a species can vary depending on behavioral state; for example Canada lynx (*Lynx canadensis*) exhibit specialist habitat selection for dense forest and high horizontal cover when foraging within their home ranges [20, 21], but will use open areas with little cover when traveling [22, 23]. Understanding these habitat selection strategies for a species across a range of behaviors can have conservation implications, since species that exhibit both specialist and generalist selection may require a narrow range of a limiting habitat element as well as more varied habitat conditions for generalist behaviors. While limiting resources are important, particularly for specialist species, research has shown that heterogenous landscapes are also often necessary [24], and can even promote population stability [25]. Thus, without an understanding of the importance of the full suite of landscape resources an animal needs across behavioral states, conservation decisions for species may be incompletely informed.

Mature forests with dense forest cover are a limiting resource that many species depend on and that are also heavily managed for human interests. Structurally complex mature forests are impacted by anthropogenic and natural disturbances, including forest thinning through management for fuel reduction to mitigate large wildfires on public lands [26], merchantable timber harvest [27], and wildfires of increasing severity and extent [28] due in large part to anthropogenic climate change and past forestry practices [29, 30]. Fishers (*Pekania pennanti*) are a forest dependent mesocarnivore species of conservation concern whose habitat is likely to be influenced by changes in forest structure as a result of past and present forest management actions, as well as natural disturbance.

Given the increasing threats to dense and mature forest in the western United States, a better understanding of the habitat needs of this species, particularly across different behavioral states which may vary in their selectivity, is critical for their conservation. Especially in the western part of their range in the US, fishers have been shown to have highly specific habitat requirements for resting and denning [31] while less is known about their moving or foraging needs, given their opportunistic diet [32, 33]. Female fishers, particularly, may be expected to have specific habitat needs to protect kits during denning, which occurs between March and April [34, 35], and to have access to adequate prey while gestating and rearing offspring [36]. Previous work suggests that fishers may not always consistently select for the same habitat elements in managed forests, depending on their behavioral state [37]. Thus, examining patterns of fisher selection for the presence of a functional response may allow us to detect differences in selectivity of resources based on behavioral state in fishers in the context of forest management.

Here, we explore the response of fishers to forest structure and other environmental characteristics primarily a result of past forest management, particularly across two different behaviors, moving (foraging) and resting. We used GPS data from 12 female fishers collected from 2013 to 2018 to evaluate resource selection in a landscape with varying amounts of past forest management, focusing on selection within a home range (third order selection; [38]). Additionally, we investigated the presence of functional responses in fisher selection of resources to determine whether fisher patterns of selectivity (i.e., specialist or generalist) differed between behavioral states. Our research questions were as follows: (1) Does fisher habitat selection vary based on behavioral state? (2) How is fisher habitat selection for each behavioral state influenced by past forest management? (3) Do fishers exhibit functional responses in selection of environmental characteristics? We predicted that fisher behavior would influence habitat selection preferences (i.e., fishers will select different characteristics when moving compared to resting), that fishers will be more tolerant to forest openings and forest structure heterogeneity when moving than when resting, and that fishers will exhibit a functional response if specialist patterns of selection are present, such as for resting behaviors that are tied to a specific range of forest characteristics.

## Study area

**Fig. 1:**
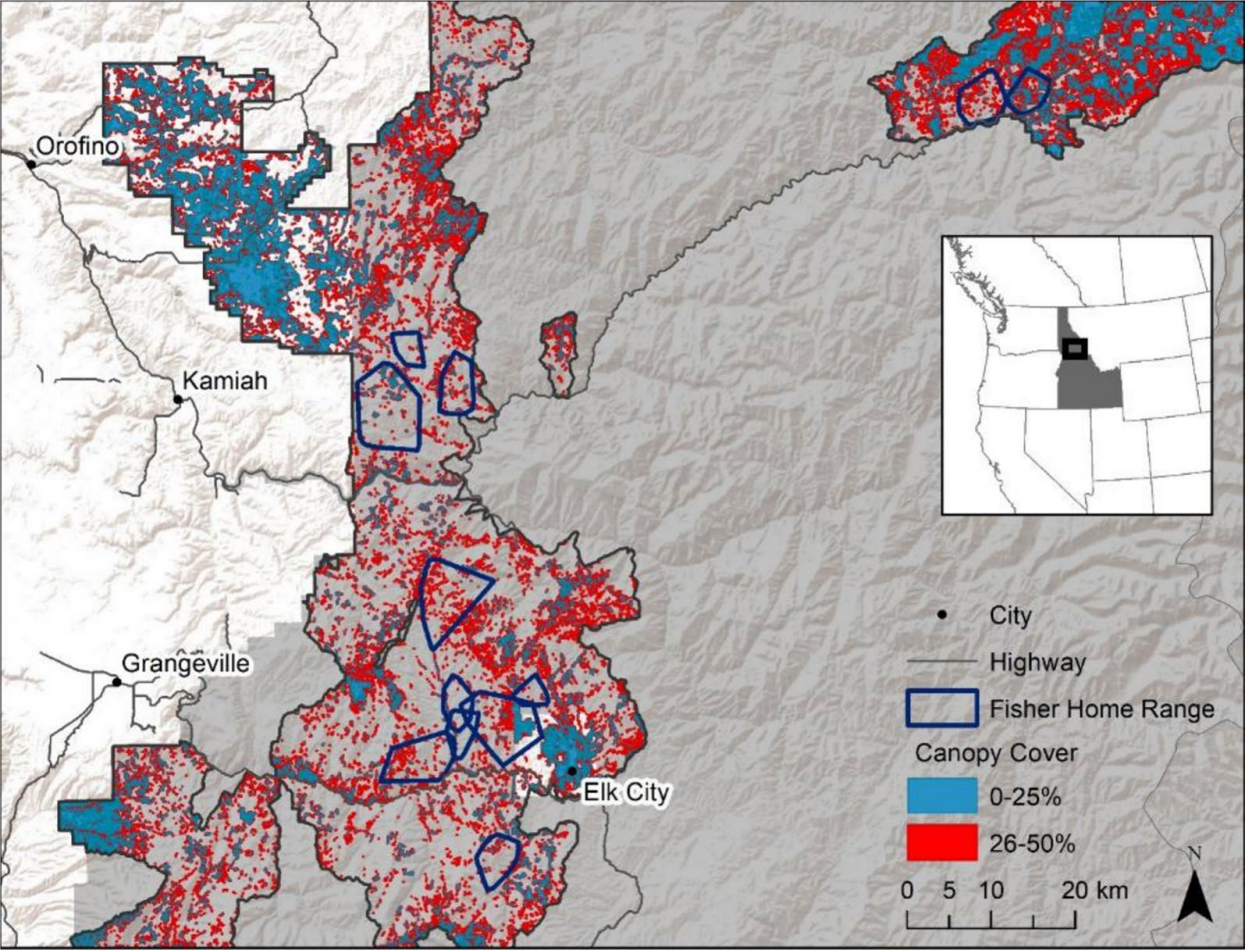
Location of the study area in the Nez Perce-Clearwater National Forests, Idaho, USA, 2013–2018. Dark gray area represents publicly owned land while light gray represents private ownership. The dark-outlined polygons show the spatial area for which LiDAR data was available and the colors indicate sparse (blue; 0–25%) and open (red; 26–50%) canopy cover, while gray-scale areas within the LiDAR footprint indicate > 50% canopy cover; dark blue polygons are fisher minimum convex polygon home ranges. Inset shows the study area relative to Idaho and the western United States

Our study focused on the Northern Rocky Mountain population of fishers in Idaho and Montana, made up of a genetic mix of native and translocated animals [39, 40]. The study area is located in northern Idaho, USA, on the Nez Perce-Clearwater National Forests, federally owned public land available for multiple uses including timber production, recreation, and wildlife habitat (Fig. 1). Elevation in the study area ranges from 400 to 2200 m, and climate is mid-latitude with warm, dry summers and cold winters [41]. Forest species composition is primarily dry-mesic mixed-conifer, dominated by Douglas-fir (*Pseudotsuga menziesii*), grand fir (*Abies grandis*), and some lodgepole pine (*Pinus contorta*), ponderosa pine (*Pinus ponderosa*), subalpine fir (*Abies lasiocarpa*) and Engelmann spruce (*Picea engelmannii*) [42]. Annual precipitation averaged 102.7 cm (range: 60–183.1 cm) per year, and temperature averaged 15.5 °C (range: 10°–20.1 °C) in summer and − 3.1 °C (range: − 7.7°–0.4 °C) in winter [43]. Snow depth on April 1, the date historically chosen to reflect total winter snowpack in the Rocky Mountains region [44], averaged 0.44 m (range: 0.08 -1.73 m) within fisher home ranges. Forest heterogeneity in the area is primarily the result of natural processes and past forest management practices; within the fisher home ranges included in this study, < 2% was affected by past wildfires (since 1950; [45]) while ~ 43% was impacted by past timber harvest (since 1950, when USFS records began to be consistently kept; [46]).

## Fisher location data

Female fishers were live-trapped in winter (Jan-Apr) from 2013 to 2018 using Havahart™ live traps that were checked daily, baited with ungulate meat, and scented with beaver and skunk scents (Idaho Fish and Game ACUC #011211). Fishers were fitted with a GPS store-on-board collar, and hand-held radio telemetry was used to periodically locate fishers to remotely download data. GPS collars were manufactured by eObs (Grünwald, Germany) and weighed ~ 69 g (< 3% of an adult female’s body weight). Collar GPS schedules were linked to the activity levels of fishers, with the frequency of GPS locations determined by fisher activity level in real time. Collars were initially programmed with three differing sets of accelerometer categories, which corresponded to likely behavior modes: slow movement (resting or denning) resulted in a GPS location every 120 min, moderate movement in a GPS location attempted every 20 min, and fast movement in a GPS location taken every 5 min [47]. Preset thresholds from a built-in tri-axial accelerometer (accelerometer settings: 18.74 Hz in a 3.5-s burst every 3 min) were used to distinguish these movement states, with threshold values taken from Brown et al. [49] (resting locations < 6400 and fast movement > 48,000), and general threshold-based sampling following [48–50]. Tri-axial accelerometers record animal movement along 3 directions (forward/backward, left/right, up/down) and measurements are unitless unless calibrated and converted to actual acceleration (m/s^2^); for this study we did not convert our measurements but used them simply to determine thresholds for movement categories. Unfortunately, for 5 collars the intermediate speed was not included, resulting in only slow (120-min fixes, accelerometer < 6400) or fast (5-min fixes, accelerometer > 6400) movement. Since GPS sampling frequency can affect inference on resource selection [51, 52], we standardized collar sampling frequency by subsampling all 5-min data into 20-min locations using the ‘amt’ package in R [53], which subsets location data based on desired temporal spacing (i.e., temporally thins data to produce a specified sampling frequency). While recognizing that the two collar program types may be measuring slightly different movement behaviors (i.e., fast and intermediate speed versus any speed faster than stationary), this allowed consistency in temporal resolution across collars and defined our ‘movement’ behavior dataset as 20-min locations from all collars. We used points collected every 115–120 min to represent ‘resting’ locations.

Additionally, we examined the fix success rate of GPS collars (the percent of successful GPS locations based on the collar schedule) since dense forest or cavity nesting behavior can obscure the sky and prevent GPS collars from determining a location [54]. The fix success rate of our collars was low (mean 50%, range: 27–80%), and therefore we performed an analysis to determine whether missed fixes were due to habitat conditions which could bias our resource selection results (see Appendix 1 for full analysis methods and results). Based on the results of this analysis, the missed fixes did not appear to be influenced by environmental conditions but instead depended more on the individual fisher (Table 4). Thus, we felt confident in proceeding with our habitat selection analyses and concluding that fix success was not consistently affected by environmental heterogeneity and therefore unlikely to bias results.

## Environmental covariates

We selected covariates based on environmental characteristics known to be important to fishers [55–57] including forest composition, structure, and topography, specifically as these relate to aspects of past forest management such as forest openings or edges (Table 5). We included Existing Vegetation Type (EVT), a remotely sensed categorical landcover layer, as measured by Landfire v1.4.0 [42], based on satellite imagery from 2013 to 2014. Based on fisher GPS locations, we selected the most prevalent EVT categories and converted them into single category binary variables (i.e., EVT category present or not) and then into proportions by taking the mean of all raster cells within a given circular neighborhood around each cell. This resulted in five EVT covariates, including mesic montane mixed conifer forest, dry-mesic montane mixed conifer forest, subalpine mesic-wet spruce-fir forest, riparian forest, and dry-mesic montane Douglas-fir forest. We considered topographic features including elevation, linearized aspect [58], slope, and topographic position index, an index of the relative elevation of an area compared to its surroundings, with negative values indicating drainages and positive values indicating ridges [59].

LiDAR-derived covariates calculated using FUSION software [60] were used to represent fine-scale characteristics of forest structure and canopy cover. Existing LiDAR (light detection and ranging) metrics from Fekety et al. [61] used in this study included canopy relief ratio, standard deviation of LiDAR heights, 25th and 50th LiDAR height percentiles, percent returns above 1 m, proportion of returns below 2 m, proportion of returns above 20 m, and horizontal cover. The USDA Northern Region Geospatial Group provided LiDAR-derived canopy cover and canopy height products. Additionally, we included aboveground biomass estimates generated from LiDAR, Landsat, topographic, and climate variables [62]. LiDAR data were collected from 2009 to 2016; since fisher locations were collected from 2013 to 2018, some locations occurred after a new natural or anthropogenic disturbance had changed the forest characteristics from the measured LiDAR conditions. To prevent error, we determined the location of disturbances that occurred between the time of LiDAR collection and fisher use of an area using a time series analysis of Landsat-derived normalized burn ratio using the LandTrendr algorithm [63]. We discarded any fisher points in these areas from the analysis (few points were discarded: 40 out of 4425 for the 20 min dataset, 0 points for the 120 min dataset). For all covariates, we considered three spatial scales to allow for differences in the scale of selection across different movement behaviors [64, 65]. We based our scales on distances determined by the 10 min, hourly, and daily average fisher movement distances (small = 100 m, medium = 400 m, and large = 4000 m scale, respectively), to match fisher selection at the home range scale.

**Fig. 2:**
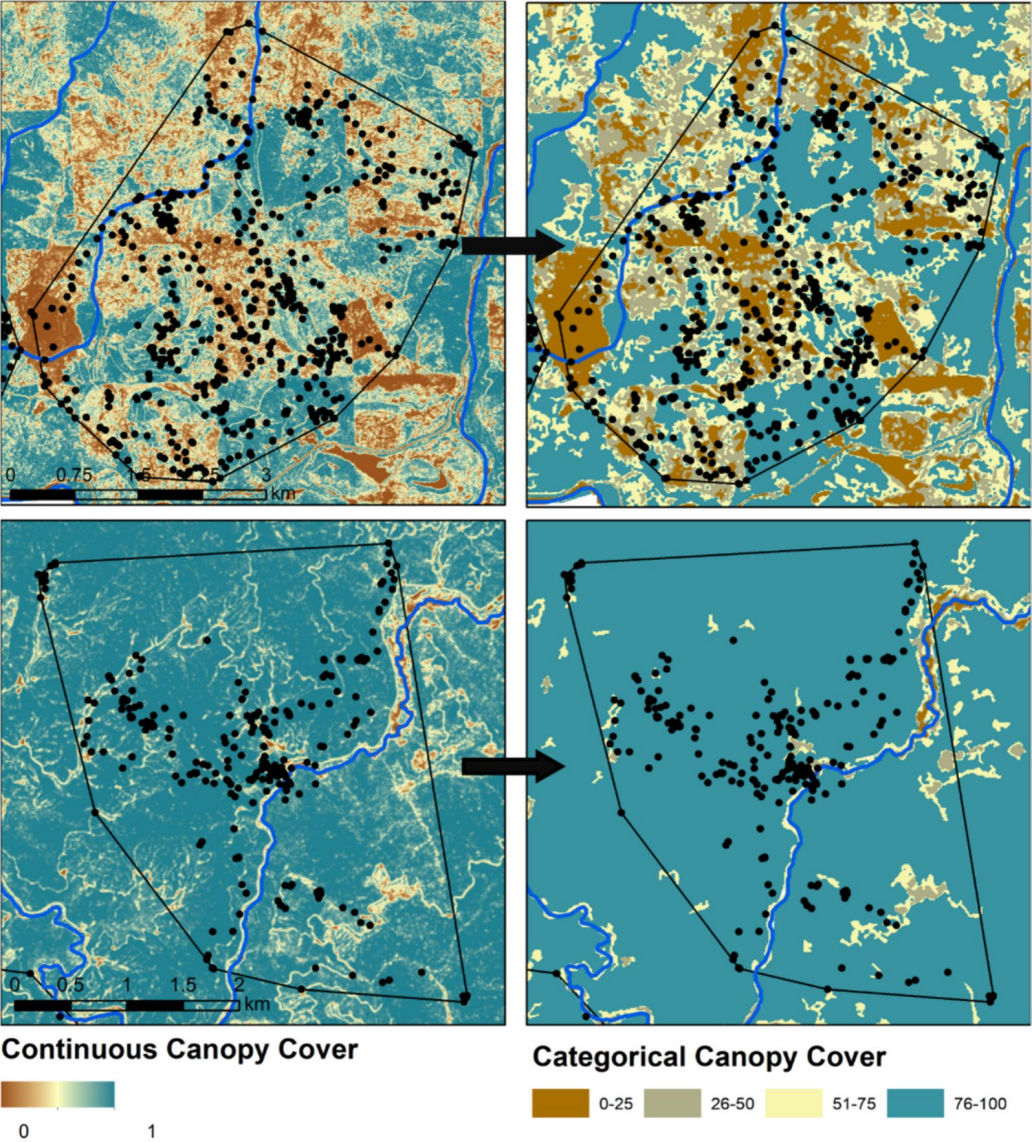
Example of the amount of forest management within a fisher home range (black polygon), from the fisher with the least amount of dense canopy cover (top panels) to the most amount of dense canopy cover (bottom panels) in northern Idaho, 2013–2018. Fisher GPS locations are represented by black dots. This graphic also illustrates the conversion of a continuous metric of canopy cover (left) into a categorical polygon-based depiction (right; colors indicate sparse: ≤ 25%, open: 26–50%, moderate: 51–75%, and dense: > 75%, respectively) to allow delineation of open patches and calculation of patch size, distance to edge, and patch edge density

To create covariates relevant to the structure and heterogeneity resulting from past forest management and natural forest openings, we created a polygon-based depiction of forest opening patches using a continuous canopy cover covariate from the LiDAR data at 10 m resolution (Fig. 2). First, we split canopy cover into four categories based on quartile thresholds (≤ 25%, 26–50%, 51–75%, > 75%). Next, we performed a neighborhood smoothing approach to remove small clusters or individual raster cells that were different from their surrounding matrix to reduce error and generate forest patches consistent with forest management actions; we used focal statistics to calculate the mode of a 30 m^2^ neighborhood around each cell and then repeated this process on the resulting raster. Finally, we converted the raster to polygons and discarded polygons that were less than 0.4 ha in size, resulting in polygons that represented four categories of canopy cover: sparse (0–25%), open (26–50%), moderate (51–75%), and dense (76–100%; see Fig. 2 for visual example). We used these polygonised patches to create four covariates related to forest openings: categorical patch canopy cover, distance to patch edge, patch size, and patch edge density (as an index of forest fragmentation, with low patch edge density equal to low forest fragmentation). We calculated patch edge density by converting polygons to lines and calculating distance of lines per unit area at each of our three spatial scales.

## Third order selection: resource selection function

To address our first question, that fishers will base their habitat selection preferences on different forest stand characteristics when moving compared to resting, we carried out separate third order resource selection functions (RSF; [66]) to evaluate habitat selection within a home range, for each behavior. For movement locations, we used the 20 min dataset (see Fisher Location Data above for further details). For resting locations, we selected locations with sampling intervals of 120 min (± 5 min), which indicated slow or stationary locations based on collar accelerometers. We created home ranges for fishers using 100% minimum convex polygons and buffered home ranges by 5 km (based on mean 24-h fisher movement distance: 4.6 km, IQR = 2.1–7.4 km) to avoid edge effects and ensure an adequate availability sample [67]. We sampled available locations for each fisher within home ranges, with both moving and resting availability samples chosen from the same pool of random locations. We based the number of available locations on the number of fisher locations, in a ratio of 1:20.

For each behavior dataset, moving or resting, we then fit a set of candidate models chosen to test various hypotheses related to forest structure and composition to determine whether differences in selection were present between behaviors at the home range scale. We first ran univariate models to determine the most predictive scale (small, medium, or large) for each covariate and calculated pairwise correlation coefficients to prevent any covariates with |r|> 0.6 from occurring in the same model. We constructed candidate models along the following hypotheses: (1) *abiotic:* topographic features are most predictive of fisher selection, (2) fishers base their habitat selection on *riparian forest* features including large trees, canopy cover, horizontal cover, species composition, and topographic drainages, (3) fisher selection is most influenced by forest *species composition*, (4) fisher habitat selection is most influenced by *forest openings*, (5) *patch edge* related features are most important to fishers, (6) *forest structure* covariates are most predictive of fisher habitat selection (see Table 1 for specific covariates and scales included in each model).

**Table 1:** Model selection table for moving and resting female fisher resource selection functions (RSF) for six candidate models based on likely environmental hypotheses

Hypothesis	Model covariates	K	AIC_c_	ΔAIC_c_	w	LL
Moving models						
Species Comp	EVT3045_400_ + EVT3047_4k_ + EVT3056_400_ + EVT3159_4k_ + EVT3227_4k_	7	33,731.64	0	1	− 16,858.8
Riparian forest	EVT3159_4k_ + TPI_4k_ + CanopyCover_400_ + Strata20_400_ + HorizontalCover_100_	7	33,740.67	9.03	0	− 16,863.3
Abiotic	Slope_400_ + TPI_100_ + Elevation_100_ + LinearAspect_4k_	6	34,035.89	304.25	0	− 17,011.9
Patch Edges	EdgeDensity_4k_ + CanLevel*DistanceToEdge + HorizontalCover_100_ + CanopyHeight_400_ + HtStd_4k_	13	34,127.48	395.84	0	− 17,050.7
Openings	EdgeDensity_4k_ + CanLevel + Pct1st_400_ + PatchSize	8	34,128.22	396.58	0	− 17,056.1
Structure	HorizontalCover_100_ + AGB_4k_ + Pct1st_400_ + StrataBelow2_400_ + Strata20_100_	7	34,180.25	448.61	0	− 17,083.1
Null	Null	2	35,583.88	1852.24	0	− 17,789.9
Resting Models						
Riparian Forest	EVT3159_4k_ + TPI_4k_ + CanopyCover_400_ + Strata20_400_ + HorizontalCover_100_	7	4266.53	0	1	− 2126.26
Structure	HorizontalCover_100_ + AGB_4k_ + Pct1st_400_ + StrataBelow2_400_ + Strata20_100_	8	4287.97	21.44	0	− 2135.98
Abiotic	Slope_400_ + TPI_100_ + Elevation_100_ + LinearAspect_4k_	6	4352.78	86.25	0	− 2170.39
Openings	EdgeDensity_4k_ + CanLevel + Pct1st_400_ + PatchSize	8	4395.44	128.91	0	− 2189.71
Species comp	EVT3045_400_ + EVT3047_4k_ + EVTt3056_400_ + EVT3159_4k_ + EVT3227_4k_	7	4455.17	188.64	0	− 2220.58
Patch edges	EdgeDensity_4k_ + CanLevel*DistanceToEdge + HorizontalCover_100_ + CanopyHeight_400_ + HtStd_4k_	12	4512.54	246.01	0	− 2244.26
Null	Null	2	4772.11	505.58	0	− 2384.05

EVT3045: Northern Rocky Mountain Dry-Mesic Montana Mixed Conifer Forest

EVT3047: Northern Rocky Mountain Mesic Montane Mixed Conifer Forest

EVT3056: Rocky Mountain Subalpine Mesic-Wet Spruce-Fir Forest and Woodland

EVT3159: Rocky Mountain Montane Riparian Forest and Woodland

EVT3227: Dry-mesic Montane Douglas-fir Forest

TPI: Topographic Position Index

Strata20: Proportion LiDAR returns above 20 m

CanLevel: Categorical canopy cover

HtStd: Standard deviation of LiDAR height above ground

Pct1st: Percent of LiDAR first returns above 1 m

AGB: Aboveground biomass

StrataBelow2: Proportion of LiDAR returns below 2 m

For each model, the covariates included, number of model parameters (K), AIC_c_, ΔAIC_c_, AIC_c_ model weight (w), and the log likelihood (LL) are given. The best-performing spatial scale for the neighborhood at which the covariates were evaluated (100m, 400m, 4000m) is given in subscript for each covariate

To address our second question, how fisher habitat selection for each behavioral state is influenced by past forest management, we performed a similar resource selection function analysis, but included all fisher locations (i.e., both moving and resting locations combined). We then tested whether fisher selection of forest structure elements related to past forest management changed depending on movement state. We fit four models, one for each past forest management metric (categorical canopy cover, patch edge density, patch size, and distance to patch edge) and included movement state (resting vs. moving) as an interaction term. For the patch size and distance to patch edge models, we fitted three-way interactions with movement state and categorical canopy cover, as fisher selection for these forest structure metrics may vary depending on the level of canopy cover in which they occur (i.e., larger patch sizes of low levels of canopy cover may be avoided, while larger patch sizes of high levels of canopy cover may be selected).

For all RSF models we used generalized linear mixed models (GLMM) with a binomial distribution and individual as a random intercept to account for repeated sampling of individuals [68]. We fit models using the ‘glmmTMB’ v.1.1.2.3 package [69] and compared model fit using the ‘AICcmodavg’ v.2.3-1 package [70] in program R and selected the top model based on AIC_c_. We standardized all covariates by dividing by the mean and subtracting the standard deviation to facilitate comparison of model coefficients.

## Functional response

We calculated functional responses to environmental characteristics considered in RSF models and past forest management metrics for both moving and resting fisher behaviors to address our third question. To calculate a functional response, we used linear models to test whether the relationship between use and availability of a given covariate for all fishers remained proportional across the range of availability in fisher home ranges. We used log of mean use as the dependent variable with log of mean availability as the predictor, and individuals as data points (n = 12) following Holbrook et al. [71] and Mysterud and Ims [18]. We calculated 90% confidence intervals for modeled intercept and slope and inferred a functional response to be present when slope was > 1, indicating increasing use as availability increased, or < 1, indicating decreasing use as availability increased. We inferred proportional habitat use equal to availability (i.e., generalist selection, no functional response) when the slope = 1 and the intercept = 0 [21, 71]. We plotted functional responses as the mean use versus mean availability to allow data visualization without the log scale.

## Results

We captured 12 fishers from 2013 to 2018; collars recorded locations from Jan to Apr, for a median 27 days (range: 9–69) and a total of 12,704 GPS locations (median locations per individual: 767, range: 348–4446). Of these, 9257 locations were collected at the 5-min or 20-min ‘movement’ threshold and 593 were ‘resting’ locations collected at the 120-min threshold. Median fisher minimum convex polygon home range size (not including the 5 km buffer for analysis) was 22.4 km^2^ (range: 9.3–62.4 km^2^); average movement distance for 5 min movement steps was 78 m (Interquartile Range (IQR) = 25–149 m), hourly movement distance was 370 m (IQR = 55–978 m), and 24-h daily movement distance was 4558 m (IQR = 2148–7374 m). Fisher home ranges contained abundant dense canopy cover (median: 79.0%, IQR: 66.1–81.8%) and low amounts of sparse (median: 2.6%, IQR: 1.8–7.4%), open (median: 4.3%, IQR: 3.7–7.1%), and moderate canopy cover (median: 15.9%, IQR: 12.6–20.4%; see Fig. 2 for examples).

## Third order RSF

In agreement with our first prediction, that fishers will base their habitat selection preferences on different characteristics depending on their behavior, we found differences in fisher habitat selection when moving versus resting. When moving (20 min locations, n = 4425), the most supported model was that corresponding with the *species composition* hypothesis (Table 1; selected spatial scales given in subscript for each covariate), with the strongest effects (as indicated by magnitude of model coefficients) those of selection for mesic montane mixed conifer forest (Table 2).

**Table 2:** Moving and resting RSF model results; parameters for the best-performing generalized linear mixed model (GLMM) of female fisher movement and resting habitat selection

	β	Std. Error	Lower 95%	Upper 95%
Model Covariate				
Intercept	− 3.31	0.25	− 3.80	− 2.81
Dry-Mesic Montane Mixed Conifer_400_	0.29	0.03	0.24	0.34
Mesic Montane Mixed Conifer_4k_	0.88	0.05	0.79	0.97
Subalpine Mesic-Wet Spruce-Fir Forest_400_	− 0.35	0.04	− 0.43	− 0.26
Montane Riparian Forest_4k_	0.31	0.03	0.25	0.36
Dry-mesic Montane Douglas-fir Forest_4k_	− 0.09	0.03	− 0.16	− 0.03
Resting Model				
Intercept	− 3.36	0.20	− 3.75	− 2.97
Montane Riparian Forest_4k_	0.51	0.07	0.38	0.64
TPI_100_	− 0.46	0.04	− 0.54	− 0.39
CanopyCover_400_	1.20	0.10	1.00	1.39
Strata20_400_	− 0.25	0.06	− 0.37	− 0.12
HorizontalCover_100_	− 0.05	0.06	− 0.16	0.06

Standardized model coefficients (β), their standard error, and 95% confidence intervals are reported; coefficients were considered significant if confidence intervals did not include 0. The best-performing spatial scale for the neighborhood at which the covariates were evaluated (100m, 400m, 4000m) is given in subscript for each covariate

When resting (120 min locations, n = 593), features reflecting the *riparian forest* hypothesis were the most supported (Table 1; selected spatial scales given in subscript for each covariate), with canopy cover contributing the most to the model (Table 2). Fishers at resting locations selected areas with greater percentage of montane riparian forest and woodland, greater percent canopy cover, lower proportion of LiDAR returns above 20 m (i.e., tall trees), and lower TPI values, indicating selection for drainages.

**Fig. 3:**
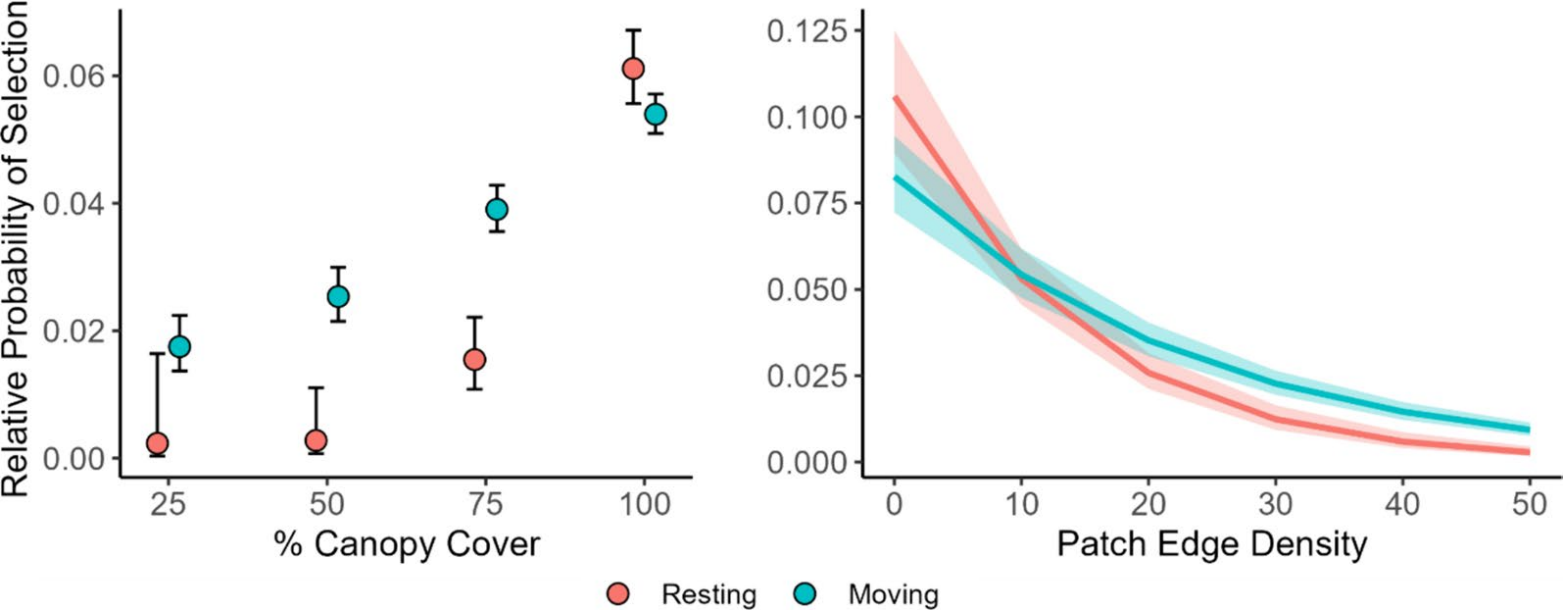
Plots show predicted results of a generalized linear mixed model of fisher relative probability of selection in response to an interaction between moving versus resting behavior and percent canopy cover (left) or forest fragmentation (patch edge density, km/km^2^, right). Fishers select higher forest cover in general and when resting compared to moving, and lower forest fragmentation in general and when resting compared to moving

In support of our second prediction, that fishers will be more tolerant to forest openings and forest structure heterogeneity when moving than when resting, we found a significant effect of moving versus resting for categorical canopy cover and forest fragmentation (patch edge density). For moving fishers, 81.3% of locations were in dense (> 76%) canopy, 13.2% in moderate (51–75%) canopy, 3.4% in open (26–50%) canopy, and 1.5% in sparse (0–25%) canopy. Of all resting locations (n = 593), 94.4% were in forest patches with dense canopy cover, with only 5.0% (30 locations) in moderate canopy patches and < 1% in patches with open or sparse canopy (1 location 0–25%, 2 locations 26–50%; see Table 6 for a summary of past forest management metrics at fisher used locations). Based on GLMMs, resting fishers were more likely than moving fishers to select dense canopy, while moving fishers were more likely than resting to select moderate, open, or sparse canopy (Table 3, Fig. 3); all fishers selected for greater canopy cover in general. Similarly, resting fishers were more likely to select low patch edge density (lower forest fragmentation) while moving fishers were more likely to select higher patch edge density (greater forest fragmentation; Fig. 3); in general, however, all fishers selected lower fragmentation. There were no consistent differences between moving and resting behaviors and fisher selection for patch size (Table 3). Compared to dense (> 76%) canopy (which we set as the reference level and in which fishers showed selection for larger patch size), fishers selected smaller patches in all lower levels of canopy cover. Resting and moving fishers did not differ in their patch size selection except in moderate (51–75%) canopy compared to dense (> 76%) canopy, with greater selection by moving fishers for small patch sizes in moderate canopy (Table 3, Fig. 6); however, low sample size of resting fisher locations in open (26–50%) and sparse (≤ 25%) canopy cover made differences in these categories difficult to detect (Table 3). Fisher selection for distance to patch edge differed between moving and resting behaviors, with moving fishers more likely to be nearer to edges than resting fisher, and for canopy cover categories, with fishers more likely to be near edges in moderate, open, or sparse canopy and far from edges in dense canopy. However the three-way interaction was not supported and differences in distance to patch edge between canopy cover categories for moving and resting locations were not detectable (Table 3), again likely due to small sample size in lower canopy cover categories (Table 3, Fig. 6).

**Table 3:** Model results of generalized linear mixed models (GLMM) of female fisher habitat selection as the result of an interaction between movement and resting behavioral states and past forest management related covariates (canopy cover, patch edge density, patch size, and distance to patch edge)

	β	Std. Error	Lower 95%	Upper 95%
Canopy Category				
Intercept	− 2.86	0.03	− 2.92	− 2.80
CanLevel25	− 1.16	0.13	− 1.41	− 0.91
CanLevel50	− 0.78	0.09	− 0.95	− 0.62
CanLevel75	− 0.34	0.05	− 0.43	− 0.25
Resting	0.13	0.05	0.04	0.23
CanLevel25:Resting	− 2.16	1.01	− 4.14	− 0.18
CanLevel50:Resting	− 2.37	0.71	− 3.77	− 0.97
CanLevel75:Resting	− 1.08	0.19	− 1.46	− 0.70
FisherID Random Intercept	0.08		0.04	0.15
Patch Edge Density				
Intercept	− 3.01	0.07	− 3.15	− 2.87
EdgeDensity_400_	− 0.43	0.02	− 0.47	− 0.40
Resting	− 0.12	0.05	− 0.23	− 0.02
EdgeDensity_400_:Resting	− 0.28	0.06	− 0.40	− 0.17
FisherID Random Intercept	0.24		0.15	0.36
Patch Size				
Intercept	− 2.94	0.09	− 3.11	− 2.77
PatchSize	0.25	0.03	0.18	0.31
CanLevel25	− 41.64	12.13	− 65.42	− 17.86
CanLevel50	− 66.05	13.07	− 91.66	− 40.44
CanLevel75	− 5.52	0.72	− 6.94	− 4.10
Resting	0.11	0.05	0.01	0.22
PatchSize:CanLevel25	− 33.34	9.88	− 52.69	− 13.98
PatchSize:CanLevel50	− 53.41	10.62	− 74.23	− 32.59
PatchSize:CanLevel75	− 4.64	0.60	− 5.82	− 3.46
PatchSize:Resting	0.07	0.07	− 0.06	0.20
CanLevel25:Resting	− 1684.00	2938.00	− 7442.27	4075.18
CanLevel50:Resting	18.01	89.38	− 157.17	193.19
CanLevel75:Resting	3.94	1.31	1.37	6.51
PatchSize:CanLevel25:Resting	− 1363.00	2381.00	− 6030.19	3303.80
PatchSize:CanLevel50:Resting	16.48	72.69	− 125.98	158.94
PatchSize:CanLevel75:Resting	4.17	1.12	1.99	6.36
FisherID Random Intercept	0.29		0.19	0.46
Distance to Edge				
Intercept	− 2.91	0.04	− 2.99	− 2.82
CanLevel25	− 2.78	0.59	− 3.94	− 1.62
CanLevel50	− 4.01	1.00	− 5.97	− 2.05
CanLevel75	− 1.16	0.35	− 1.86	− 0.47
DistToEdge	0.18	0.01	0.15	0.21
Resting	0.10	0.05	0.00	0.20
CanLevel25:DistToEdge	− 2.78	0.88	− 4.50	− 1.07
CanLevel50:DistToEdge	− 4.69	1.38	− 7.39	− 2.00
CanLevel75:DistToEdge	− 1.40	0.50	− 2.38	− 0.42
CanLevel25:Resting	− 25.02	31.39	− 86.55	36.52
CanLevel50:Resting	− 27.07	25.96	− 77.94	23.80
CanLevel75:Resting	− 2.19	1.71	− 5.54	1.16
DistToEdge:Resting	0.08	0.04	0.00	0.15
CanLevel25:DistToEdge:Resting	− 31.05	41.21	− 111.82	49.73
CanLevel50:DistToEdge:Resting	− 32.94	33.87	− 99.33	33.44
CanLevel75:DistToEdge:Resting	− 1.71	2.39	− 6.40	2.98
FisherID Random Intercept	0.14		0.08	0.22

Standardized model coefficients (β), their standard error, and 95% confidence intervals are reported; coefficients were considered significant if confidence intervals did not include 0

**Fig. 4:**
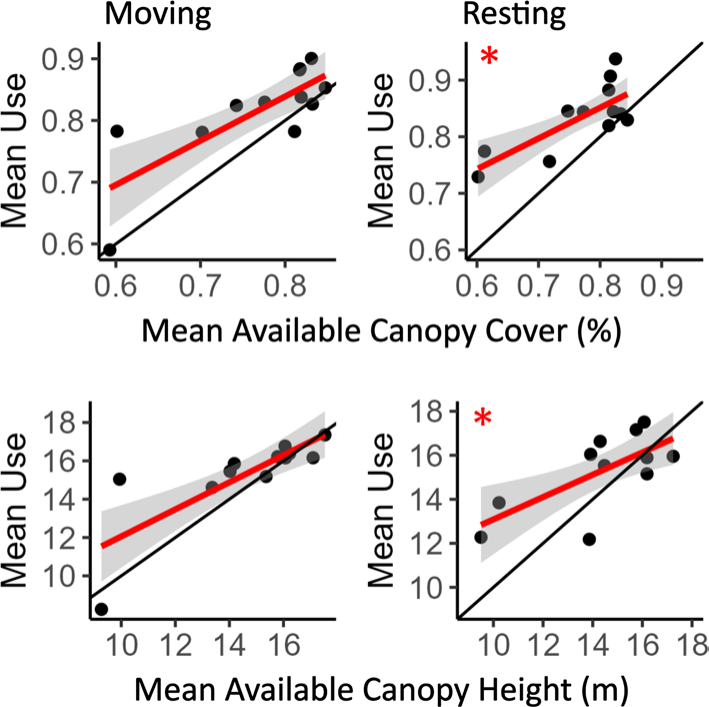
Plots show results of a linear model of average use versus average availability across fishers (each data point represents an individual), i.e,. a functional response analysis. Results indicate a generalist pattern of selection in moving fishers (left) and a specialist pattern of selection in resting fishers (right) for two habitat covariates, percent canopy cover (top) and canopy height (bottom). Black lines indicate hypothetical proportional use (intercept = 0, slope = 1), red lines show modeled fisher results, gray shaded areas are 90% confidence intervals, and red asterisks indicate significant deviation from proportional use. For moving fishers, use of habitat covariates remains proportional to their availability as availability changes, while resting fishers show greater use (points above the black line) of covariates when at low availability which decreases to proportional use (points on the black line) at higher availability. See Table 7 for all functional response results

## Functional response

We found differences between moving and resting fishers in the presence of functional responses for habitat-related covariates. For moving fishers, we found proportional use of all covariates and no evidence of functional response, indicating a generalist response to all habitat related covariates (Fig. 4; Table 7). For fishers at rest, however, several covariates were used at greater proportions when their availability was low, indicating a specialist functional response (Fig. 4; Table 7; [71, 72]). When resting, fishers used patches with taller trees, greater canopy cover, and lower TPI values at greater proportion when these resources were less available and decreased their use to proportional as availability increased, consistent with a specialist functional response (Table 7). Fisher selection of covariates related to openings and heterogeneity did not change as their availability changed, however. For moving and resting fishers, we found no consistent significant deviation from use proportional to availability as availability of patch canopy cover, patch edge density, mean patch size, or distance to patch edge changed across home ranges (Table 7). Resting fishers had too few points in the low canopy cover categories (open and sparse) to calculate functional responses for these groups (Table 7).

## Discussion

Identifying and maintaining the resource needs of vulnerable species is one of the cornerstones of conservation biology, but variation in those needs depending on an individual’s behavior is often overlooked. The resource requirements of fishers when resting and denning are narrow and consequently well-studied; ideal foraging conditions are less defined [37], particularly in the Rocky Mountain population of fishers. Our work supports the idea that female fishers are both a habitat specialist and a habitat generalist; resting and denning behaviors require a more specific set of conditions while foraging habitat requirements are more general. When resting, fishers selected forest characteristics related to composition and structure, while only broad vegetation type was most predictive of fishers when moving. Resting fishers selected more contiguous forest and stayed farther from patch edges, and both moving and resting fishers avoided patches with lower canopy cover, although this avoidance was stronger for resting individuals. Response to forest characteristics associated with past forest management, such as patch edges, forest fragmentation, or open patches, did not change as their availability changed, indicating a possible tolerance for early seral forest structure among foraging fishers despite their known dependence on mature forests [55].

A probable mechanism for the specialist/generalist pattern we detected is that fishers are dietary generalists [32, 33, 73, 74, 108] while also preferring structurally complex forest for denning and resting. This pattern of selection may be an attempt to maximize a tradeoff: while mature forests with dense canopy and little understory offer resting and denning opportunities, these forest stands are often lacking in prey availability. In contrast, densities of prey species are often high in openings that have recently been disturbed (e.g., chipmunks and small mammals; [75]) or in regenerating forest stands with dense saplings (e.g., snowshoe hares; [76, 77]). The fishers in our study were monitored from January through April, an important period leading up to and including denning and likely parturition [34, 36], and a season often characterized by lower prey availability, when successful foraging was likely to be particularly important to female fishers. Selection differences in fishers based on movement or resting behavior are supported by previous work, with multiple studies confirming strong selection for large trees with cavities when denning or resting [31, 55, 78], while foraging habitat is less limiting and less well characterized [37]. For example, West Coast fisher population studies indicate that fishers use a broad range of tree sizes and ages [37].

Like previous studies, we found that resting fisher habitat was best predicted by covariates related to forest structure at the stand scale. Resting fishers selected areas with greater canopy cover, more riparian habitat, and arranged in topographic drainages, all characteristic of the type of “classic” fisher habitat (i.e., large, wet, decaying trees) reported throughout the literature [31, 55, 79] in the western portion of fisher range. While moving, however, the more general forest species composition characteristics were most predictive of fisher habitat; fisher presence was most strongly predicted by mesic montane mixed conifer forest, while also exhibiting a preference for riparian forest and an avoidance of spruce-fir forest and drier Douglas-fir forest. The Northern Rocky Mountain fisher population is not well studied, and habitat selection has generally been assessed at the home range level or larger, without consideration for differences in fisher behavior. Thus, strong selection for resting and denning habitat, such as mature forest [56], large diameter trees [55], and mesic forest with tall trees [80], has been detected across multiple studies, while the more generalist selection signature of moving fishers may be swamped by this signal and go undetected. Sauder and Rachlow [57], however, found that fisher core areas in this population were characterized by greater heterogeneity of patch edges and canopy cover, indicating a preference for a more general array of forest characteristics in high use areas. Due to the movement thresholds of our collars, the movement state in our study is also a more general category than resting, potentially encompassing activities including foraging, territorial patrolling, and traveling between high use areas, the combination of which may contribute to the generalist signature.

Interestingly, we found no evidence of a functional response for moving female fishers in any of the habitat or disturbance covariates we examined; for instance, while fishers avoided forest patches with < 75% cover while moving, avoidance stayed consistent regardless of how much of a fisher’s home range consisted of this more open canopy. Functional responses are often detected in response to disturbance-related covariates, with responses to a given resource changing depending on how much of the resource an individual is exposed to [18, 71]. For instance, cougars (*Puma* *concolor*) showed decreased avoidance of certain anthropogenic disturbances when their home ranges were more disturbed [14], presumably making the ‘best of a bad job’ by selecting for the least negative impact in a sub-optimal home range [81]. Alternatively, especially preferred resources may always be selected disproportionately above their availability, such as Canada lynx which consistently select for certain forest types even when availability is high [82]. Selection differences depending on availability are especially important in the case of habitat disturbances, such as decreased forest structure and canopy from forest management [83] or encroaching human development [14], since individuals may seem tolerant to disturbance until it reaches a certain threshold [84].

There are several factors which may contribute to the lack of functional response to past forest management covariates. First, use in proportion to availability is a characteristic of generalist selection, in which animals are behaviorally flexible enough to make a living out of whatever resources are at hand. There is some support for this hypothesis in the literature, in which foraging fishers have been noted to have more general habitat use and broader prey selection, even eating a surprising amount of alternative prey sources [33]. Carnivores in general are often habitat generalists, capable of altering their habitat selection to take advantage of shifting prey populations [85]. Second, habitat selection is hierarchical, with fine-scale habitat choices limited by the larger-scale choices already made through population and home range placement choices [38]. For fishers in our study, the amount of disturbed habitat in home ranges was low (average of ~ 7% with < 50% canopy cover), indicating that selection may have already taken place at a coarser scale, resulting in universally low availability of disturbed habitat at a level that fishers can tolerate, or a range of disturbed habitat availability that is too narrow to detect a functional response. Our trapping experience supports this, anecdotally, as a primary goal of this study was to examine fisher habitat across a range of managed forests, but we were unsuccessful in trapping any fishers in areas with a high proportion of past disturbance. Finally, foraging fishers may be more tolerant of forest openings and heterogeneity from past management than expected, and willing to trade the risk of using more open canopy for the increase in prey associated with the greater ground cover and understory of early successional stands [75]. Smith [86] found that fishers in Oregon continued to use recently thinned habitat in their home ranges given that sufficient den sites and canopy cover > 50% was maintained within 2 km in the home range, Green et al. [87] showed that fishers translocated to an area in the Sierra Nevada of California with high levels of current and historical forest management survived and increased in abundance over 7 years of monitoring, and Niblett et al. [88] found that fisher home ranges in California could contain up to 25% of open canopy provided that enough large trees for den locations were present.

The consideration of fisher behavior in this study allowed a more complete understanding of fisher habitat selection beyond their common perception as a highly specialized old-forest species. If we had not considered fisher movement behavior when analyzing selection patterns, we would potentially misidentify the landscape characteristics that fishers need to complete important life history stages, including foraging behavior during gestation and early denning. Since our study focused only on female fishers due to their importance in maintaining a reproductive population, caution should be used in applying these results to male fishers. While males are likely to be broadly similar in their habitat needs for resting and foraging conditions, more research is needed to determine if and how behaviors that may differ by sex, such as territorial patrolling or mate-searching, influence habitat selection in this species. Tolerance to risk is also often a sex-biased behavior, with males more willing to accept greater risk than reproductive females [89, 90], which may have implications for tolerance of past forest management as well. Our results support the consideration of behavioral state as part of RSF analyses to provide important connections to species biology and a more nuanced understanding of habitat selection. The ability to more strongly connect RSF analyses to the entirety of a species’ habitat needs will allow more biologically realistic predictions of important habitat, which in turn will allow more informed conservation management.

## Management implications

Our results indicate a multi-level response by female fishers to forest openings and forest heterogeneity from past forest management, with home ranges containing low amounts of open canopy cover and fishers also avoiding forest openings and fragmentation while both moving and resting. Fishers stayed close to patch edges and used smaller patches of forest openings when canopy cover was lower (≤ 75%), and resting locations were almost nonexistent in any forest patch with ≤ 75% cover, indicative of their likely desire to remain safe from predation when not trying to acquire food resources. In sparse (≤ 25%) canopy areas, the average patch size used by moving fishers was 2.4 ha, and the average distance from a patch edge was 9.8 m (see Table 6 for additional summaries of forest structure related to past forest management). Female fisher home ranges contained only limited amounts of low canopy cover patch openings, and the majority of fisher locations (94% of resting fisher locations and 81% of moving fisher locations) were in dense (> 76%) canopy, indicating that selection had already taken place to avoid open areas when fishers were placing home ranges. Thus, an increase in active forest management that results in large patches of open canopy, for example from thinning or clearcutting, may prove a barrier to fisher movement and a negative impact on foraging success. The lack of functional response by moving fishers in our study to increases in home range heterogeneity, however, suggest there may be some tolerance to forest openings and heterogeneity, at least at the levels of availability found within home ranges here (i.e., average of ~ 7% of home range with < 50% canopy cover). Many studies also point out the necessity of maintaining large trees, live and dead, on the landscape when performing thinning or salvage forest management [86, 91], and the results of our analysis on resting habitat selection concur. Large trees capable of providing cavities for nesting are extremely slow to form and may be declining on the landscape due to fire and timber management [92, 93]. Therefore, despite the more generalist needs of fishers when moving, the protection of large trees and areas of dense canopy when planning management actions is likely of great importance to maintain a reproductively successful population of fishers.

## Figures and Tables

**Fig. 5:**
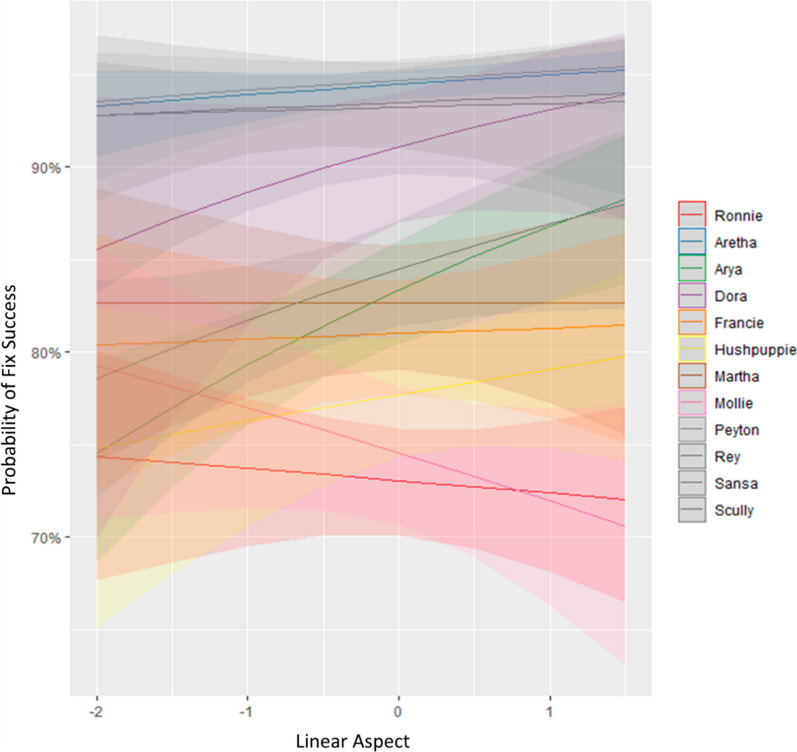
Predictions of collar fix success from the second-best performing fix success model, FisherID + linear aspect. Predicted lines show response (with 95% confidence interval) for each fisher across the range of linear aspect; inconsistent positive and negative responses indicate that linear aspect is not a good predictor of fix success across individuals

**Fig. 6:**
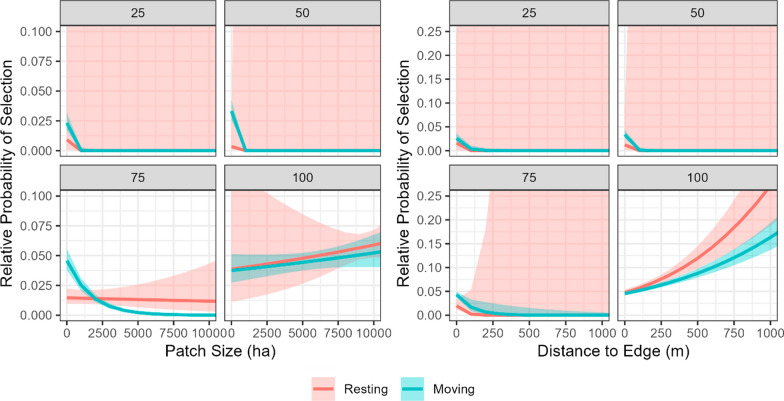
Plots show female fisher relative probability of selection based on generalized linear mixed models (GLMMS) of three-way interactions between movement type (resting or moving), canopy cover category (sparse (≤ 25%), open (26–50%), moderate (51–75%), and dense (≥ 76%)), and either patch size (left plot) or distance to patch edge (right plot). Panels in each plot show the predicted response in each canopy category, with panel titles representing the canopy level (sparse = 25, open = 50, moderate = 75, dense = 100). In dense canopy, fishers select large patches, while selecting for smaller patches in all other canopy categories; there was no difference in patch size used between moving or resting behaviors. Additionally, in dense canopy, fishers selected greater distances from patch edges compared to all other canopy levels, and resting locations were more likely to be farther from an edge than moving locations. See Table 3 for model coefficients. Small sample sizes in low canopy cover categories resulted in large 95% confidence intervals (indicated by colored shading) and lack of statistical power to detect differences in sparse and open canopy categories

## Data Availability

The datasets supporting the conclusions of this article will be available upon request to the corresponding author.

## Appendix 1

### Fix success analysis

A concern when tracking species, such as fisher, that live in dense forest and nest in cavities is that the locations from a GPS collar will be biased due to systematically missed telemetry fixes when the animal is in dense cover, and thus that conclusions from the analysis will underestimate the importance of cover-associated habitat covariates [54, 94]. To correct for habitat-related missed fix bias, one approach is to model the probability of obtaining a fix based on habitat characteristics and then to generate a predicted value of fix success at all locations; the data are then weighted by the inverse of this probability value to provide more weight to less probable locations [54, 95]. To determine if this was a problem with our data, we initially quantified the fix success of each collar, which is a measure of the number of successful GPS fixes relative to the number of attempted fixes. The fix success of our collars was poor, with a mean fix success rate of 50% (range 27–80%). Thus, we performed an analysis to determine whether the missed fixes were related to habitat characteristics and would therefore cause bias in the analysis or were endogenous to collars or individuals and therefore, while not optimal, the analysis could proceed with the existing data without habitat bias.

We used our 5-min fisher collar dataset to model the importance of environmental and endogenous sources of variation to the probability of collar fix success. Since an unsuccessful fix attempt acquires no coordinates, we estimated the location of missed fixes as equally spaced points between two successful locations, following Graves and Waller [96], and only included two or fewer consecutive missed fixes to reduce estimated location uncertainty. We used the ‘adehabitatLT’ v.0.3.25 [97] package in program R v.4.1.1 [98] to estimate the spatial location of missed fixes. At all successful and estimated missed locations, we extracted covariates we hypothesized would likely block satellite reception and influence GPS fix success, including canopy cover, aboveground biomass, horizontal cover, slope, aspect, topographic position index, and elevation. We also considered the individual fisher ID, year, and collar schedule as additional sources of variation in fix success. Using the R package ‘lme4’ v.1.1–27.1 [99], we fit generalized linear models (GLM) with a logistic link and fix success or failure as the dependent variable. We did not include a random effect since we wanted to test for the influence of fisher ID on fix success, and thus we included this as a covariate instead. We screened all covariates for pairwise correlation |r < 0.6| and did not include correlated covariates in the same model. We considered 11 candidate models (see Table 4 for model specifications) that we hypothesized were most likely to impact collar fix success, including interactions between vegetation cover-related covariates and fisher ID to account for potential differences in collar success among individuals, as well as models focused on only ID, year, or additive combinations of habitat-related covariates; we compared model performance using Akaike’s Information Criteria corrected for small sample size (AIC_c_; [100, 101]).

### Fix success analysis results

We did not find evidence that environmental characteristics were affecting the success rate of GPS collars on fishers. The model containing only fisher ID was the top-performing in our fix success candidate model selection procedure, with little model uncertainty (Table

**Table 4:** Model selection table for assessing the influence of intrinsic and environmental variation on the GPS collar fix success of 12 radio-collared female fishers (*Pekania pennanti*) in Idaho, USA, 2013–2018

Model specification	K	AIC_c_	ΔAIC_c_	w	LL
FisherID	12	7670.23	0	0.69	− 3823.1
FisherID *LinearAspect	24	7672.85	2.62	0.19	− 3812.37
FisherID + TPI_100_ + AGB	14	7673.76	3.53	0.12	− 3822.86
FisherID*AGB	24	7685.01	14.78	0	− 3818.45
FisherID *HorizontalCover	24	7685.95	15.72	0	− 3818.92
FisherID *CanopyCover	24	7688.24	18.01	0	− 3820.06
CollarType	2	7911.87	241.64	0	− 3953.93
Year	6	7992.51	322.28	0	− 3990.25
TPI100 + LinearAspect + Slope + Elevation	5	8243.51	573.28	0	− 4116.75
Slope + CanopyCover + HorizontalCover + LinearAspect	5	8255.88	585.65	0	− 4122.94
Null	1	8282.24	612.01	0	− 4140.12

For each model, the covariates included in the model, number of model parameters (K), AIC_c_, ΔAIC_c_, AIC_c_ weight (w), and the log likelihood (LL) are given. Abbreviation ‘TPI100’ indicates the covariate ‘topographic position index’ calculated at a 100m neighborhood and ‘AGB’ indicates the LiDAR variable ‘aboveground biomass’

4), indicating that variation in successful collar location attempts depended more on the individual fisher than on any external environmental features. The second-best model was > 2 ΔAIC from the top model and contained an interaction effect between fisher ID and linear aspect. A plot of predicted fix success based on this model showed an inconsistent response to aspect across individuals (Fig. 5); thus, we felt confident in inferring that fix success was not consistently affected by environmental heterogeneity and therefore unlikely to bias the results of a habitat selection analysis.

See Fig. 6 and Tables 5, 6, 7.

**Table 5:** This table provides a description of the covariates considered for inclusion in models of resource selection for fishers in our study area in Idaho, USA, 2013–2018

Covariate	Description	Units	Mean and range	Source
Elevation	Elevation above sea level	Meters	1346 (757–1894)	[106]
Linear Aspect	A transformation of circular aspect into a linear variable, with 0 = hotter dryer south-SW facing, 1 = cooler wetter N-NE facing	NA	0.54 (0–1)	[102]
Slope	Gradient of elevation	Degrees	18.4 (0–52)	[106]
Topographic Position Index (TPI)	Index of landscape concavity (drainages; negative values) or convexity (ridges; positive values)	NA	− 1.65 (− 25–31)	[106]
EVT3047	Existing vegetation type category Northern Rocky Mountain Mesic Montane Mixed Conifer Forest	Proportion	0.35 (0–1)	[105]
EVT3045	Existing vegetation type category Northern Rocky Mountain Dry-Mesic Montane Mixed Conifer Forest	Proportion	0.44 (0–1)	[105]
EVT3056	Existing vegetation type category Rocky Mountain Subalpine Mesic-Wet Spruce-Fir Forest and Woodland	Proportion	0.07 (0–1)	[105]
EVT3159	Existing vegetation type category Rocky Mountain Montane Riparian Forest and Woodland	Proportion	0.08 (0–0.90)	[105]
EVT3227	Existing vegetation type category Dry-mesic Montane Douglas-fir Forest	Proportion	0.04 (0–0.83)	[105]
Elev_p25	25th percentile of LiDAR heights above ground	Meter	8.87 (0.28–21.1)	[106]
Elev_p50	50th percentile of LiDAR heights above ground	Meter	14.2 (0.63–28.7)	[106]
Ht Std	Standard deviation of LiDAR heights above ground	Meter	7.46 (0–18.5)	[106]
Strata below 2m	Proportion of first LiDAR returns between ground level and 2.0 m	Proportion	0.21 (0.01–0.82)	[106]
Percent first returns above 1 m	Percentage of first LiDAR returns greater than 1.0 m above ground level	Percent	81.5 (0–100)	[106]
Strata 10m	Proportion of first LiDAR returns greater than 10.0 m above ground level	Proportion	0.50 (0–0.86)	[106]
Strata 20m	Proportion of first LiDAR returns greater than 20.0 m above ground level	Proportion	0.23 (0–0.69)	[106]
Canopy relief ratio	Measure of forest canopy roughness. Calculated as: ((mean—min) / (max – min))	Proportion	0.41 (0–73)	[106]
Horizontal cover	Measure of horizontal obstruction between 0 and 2 m above ground level	Proportion	0.51 (0.24–1)	[103]
Above ground biomass	Live and dead aboveground biomass of trees	Mg / ha	207 (2.12–603)	[104]
Canopy Cover	Percent canopy cover	Percent	83 (0–100)	[107]
Canopy Height	Canopy height	Meters	15.6 (0.08–49.1)	[107]
CanLevel	Polygon-based depiction of canopy cover	Categorical	25,50,75,100	[107]
Distance to edge	Distance to nearest patch edge	Meters	138 (0–911)	[107]
Patch size	Size of polygon-based patch	Hectares	9813 (0–21841)	[107]
Patch Edge Density	Forest fragmentation metric	Km/km^2^	10.2 (0.38–35)	[107]

The covariate name, description, unit of measure, mean and range, and data source are provided. The mean and range values were calculated across all fisher GPS locations collected for the study

**Table 6:** Summaries (median, 25th_,_ and 75th percentile) of amounts of patch size, distance from patch edge, and percent of used and available locations in each category of canopy cover for used (actual GPS locations) versus available (random locations in home ranges) locations for moving and resting female fishers in Idaho, USA, 2013–2018

		Canopy Level Category	Use			Avail		
			Median	25th	75th	Median	25th	75th
Moving	Patch size (ha)	25	2.4	1.7	22.3	53.4	12.2	224.2
		50	1.9	1.8	7.5	44.9	10.2	154.6
		75	97.5	27.2	138.4	210.0	120.6	474.3
		100	10,800.3	7046.0	12,374.9	10,483.0	8076.7	14,561.4
	Distance to Edge (m)	25	9.84	7.80	17.00	23.60	17.60	29.60
		50	6.05	4.82	8.33	9.92	9.64	11.00
		75	10.00	8.69	11.40	11.80	10.60	12.70
		100	110.00	60.60	150.00	129.00	103.00	138.00
	Percent of locations in canopy level patch	25	1.58	0.77	1.89	2.58	1.84	7.40
		50	1.80	1.13	3.87	4.30	3.71	7.10
		75	13.70	7.57	21.60	15.90	12.60	20.40
		100	83.60	75.30	89.50	79.00	66.10	81.80
Resting	Patch size (ha)	25	2.5	2.5	2.5	55.8	27.0	227.4
		50	33.3	17.4	49.0	53.0	11.6	173.2
		75	14.0	4.8	74.9	227.0	123.4	416.4
		100	10,996.5	5449.9	12,101.7	10,461.9	8094.1	14,236.0
	Distance to Edge (m)	25	3.45	3.45	3.45	28.80	17.90	32.80
		50	2.86	2.49	3.24	10.40	9.40	11.70
		75	9.86	4.86	14.20	12.00	11.20	12.90
		100	97.80	65.20	145.00	128.00	105.00	137.00
	Percent of locations in canopy level patch	25	1.18	1.18	1.18	2.45	1.22	6.44
		50	2.97	2.07	3.87	4.81	3.56	8.56
		75	8.71	7.43	16.00	15.20	12.70	20.10
		100	93.30	87.30	100.00	79.30	68.10	81.10

**Table 7:** 90% confidence intervals around intercepts and slopes testing functional responses to environmental covariates for resting and moving female fishers in Idaho, USA, 2013–2018

Covariate	Resting		Moving	
	Intercept	Slope	Intercept	Slope
Aboveground biomass	− 0.18–3.87	0.28–1.05	− 1.32–2.96	0.44–1.26
Canopy Cover	− 0.12–0.01	**0.25**–**0.7**	− 0.12–0.07	0.38–1.01
Canopy Height	0.38–2.05	**0.26**–**0.89**	− 0.2–1.62	0.41–1.09
Canopy Relief Ratio	− 0.6–0.03	**0.29**–**0.98**	− 0.43–0.09	0.51–1.08
Elevation	0.58–5.06	**0.29**–**0.92**	− 0.06–4.38	0.39–1
25th LiDAR Height Percentile	0.08–1.66	0.27–1	− 0.3–1.19	0.46–1.16
50th LiDAR Height Percentile	− 0.23–1.87	0.31–1.11	− 0.69–1.31	0.51–1.28
Standard Deviation of LiDAR Height	− 1.06–1.34	0.32–1.54	− 1.56–0.94	0.52–1.8
Northern Rocky Mountain Dry-Mesic Montana Mixed Conifer Forest	− 0.28–1.17	0.96–2.03	− 0.05–0.67	0.98–1.49
Northern Rocky Mountain Mesic Montane Mixed Conifer Forest	− 0.25–1.17	0.81–1.84	− 0.24–0.81	0.82–1.58
Rocky Mountain Subalpine Mesic-Wet Spruce-Fir Forest and Woodland	− 2.25–1.01	0.73–2.06	− 1.85–1.3	0.83–2.07
Rocky Mountain Montane Riparian Forest and Woodland	− 3.03–9.54	0.21–3.99	− 3.28–3.77	− 0.01–2.14
Dry-mesic Montane Douglas-fir Forest	− 2.02–3.7	0.68–2.26	− 0.96–2.42	0.82–1.76
Horizontal Cover	− 0.61–0.11	0.18–1.22	− 0.29–0.13	0.59–1.18
Linear Aspect	− 0.95–0.74	− 0.82–1.93	− 0.45–0.84	0.19–2.27
Patch Edge Density	− 4.04–1.76	0.17–2.37	− 2.1–0.51	0.71–1.7
Percent of LiDAR first returns > 1 m	0.77–3.3	**0.25–0.84**	− 0.11–2.92	0.34–1.04
Slope	− 1.08–1.16	0.33–1.35	− 1.44–0.76	0.57–1.57
Proportion of LiDAR returns < 2 m	− 1.2–0.46	0.35–1.48	− 0.65–0.87	0.66–1.7
Proportion of LiDAR returns > 10 m	− 0.42–0.06	**0.31–0.87**	− 0.33–0.35	0.51–1.29
Proportion of LiDAR returns > 20 m	− 1.47–0.5	0.04–1.29	− 1.03–0.47	0.33–1.28
Topographic Position Index	1.96–2.62	**− 0.49–0.59**	0.9–3.12	− 0.68–1.21
CanLevel25	NA	NA	− 1.66–0.72	− 0.12–1.33
CanLevel50	NA	NA	− 2.72–− 0.34	0.72–1.99
CanLevel75	− 6.44–3.64	− 0.46–2.98	− 5.7–0.55	0.69–2.92
CanLevel100	2.39–3.73	**0.18–0.5**	− 0.84–2.1	0.53–1.22
DistToEdge25	NA	NA	− 2.71–1.98	0.16–1.64
DistToEdge50	NA	NA	− 6.76–1.91	− 0.03–3.72
DistToEdge75	− 32.33–5.24	− 1.37–13.47	− 0.68–2.67	− 0.15–1.22
DistToEdge100	− 1.82–5.1	− 0.1–1.36	− 2.67–3.02	0.34–1.55
EdgeDensity25	NA	NA	− 12.76–10.92	− 2.49–4.96
EdgeDensity50	NA	NA	− 2.81–2.63	0.14–1.86
EdgeDensity75	− 5.35–1.21	0.5–2.64	− 3.12–0.52	0.75–1.96
EdgeDensity100	− 2.97–2.56	− 0.16–2.1	− 1.73–0.78	0.62–1.64
PatchSize25	NA	NA	− 4.83–2.32	0.13–1.49
PatchSize50	NA	NA	− 1.44–4.04	**− 0.37–0.76**
PatchSize75	− 13.81–5.56	− 0.21–2.55	− 3.85–3.14	0.28–1.36
PatchSize100	− 1.34–5.58	0.44–1.13	− 1.85–4.21	0.57–1.18

Proportional use of resources is demonstrated when slope is indistinguishable from 1 and intercept equals 0. Significant deviations from proportional use, i.e., a functional response, are bolded

## Abbreviations

ACUC: Animal care and use committee
GPS: Global Positioning System
EVT: Existing vegetation type
RSF: Resource selection function
AIC: Akaike information criteria
IQR: Interquartile range
USDA: United States Department of Agriculture
USFS: United States Forest Service
LiDAR: Light detection and ranging
